# An Overview of Cytomegalovirus Infection in Pregnancy

**DOI:** 10.3390/diagnostics12102429

**Published:** 2022-10-07

**Authors:** Mihaela Plotogea, Al Jashi Isam, Francesca Frincu, Anca Zgura, Xenia Bacinschi, Florica Sandru, Simona Duta, Razvan Cosmin Petca, Antoine Edu

**Affiliations:** 1 Department of Obstetrics and Gynecology, “Nicolae Malaxa” Clinical Hospital, 022441 Bucharest, Romania; 2 Faculty of Medicine, “Titu Maiorescu” University, 031593 Bucharest, Romania; 3 Department of Obstetrics and Gynecology, “Carol Davila” University of Medicine and Pharmacy, 020021 Bucharest, Romania

**Keywords:** cytomegalovirus, immunoglobulins, congenital infection, congenital CMV imaging diagnosis

## Abstract

The objective of this review was to bring to attention cytomegalovirus (CMV) infection during pregnancy, taking into consideration all relevant aspects, such as maternal diagnosis, fetal infection and prevention, prenatal diagnosis, and postnatal prognosis. A literature review was performed regarding adult and congenital infection. General information regarding this viral infection and potential related medical conditions was provided, considering the issues of maternal infection during pregnancy, transmission to the fetus, and associated congenital infection management. Prenatal diagnosis includes maternal serum testing and the confirmation of the infection in amniotic fluid or fetal blood. Additionally, prenatal diagnosis requires imaging techniques, ultrasound, and complementary magnetic resonance to assess cortical and extracortical anomalies. Imaging findings can predict both fetal involvement and the postnatal prognosis of the newborn, but they are difficult to assess, even for highly trained physicians. In regard to fetal sequelae, the early diagnosis of a potential fetal infection is crucial, and methods to decrease fetal involvement should be considered. Postnatal evaluation is also important, because many newborns may be asymptomatic and clinical anomalies can be diagnosed when sequelae are permanent.

## 1. Introduction

Cytomegalovirus (CMV) is a herpes virus with high prevalence among children and the adult population, especially in developing countries [1]. Diagnosis is rare in the general population, primarily because of the lack or mild form of clinical symptoms. Additionally, the disease does not usually require treatment in the immunocompetent population. However, early diagnosis and proper management are crucial in immunosuppressed patients during pregnancy and in the postnatal period [2]. If primary CMV infection occurs periconceptionally or in the first trimester of pregnancy, it can impact fetal development and result in severe abnormalities, known as a congenital CMV infection [3]. The diagnosis of both maternal and fetal infections is often a challenge and can be established directly or indirectly. The serum testing of the mother is highly important and can predict fetal infection [4]. The direct detection of CMV from the amniotic fluid of fetal blood may put the fetus at risk, while imaging findings are not pathognomonic for CMV fetal infection. Most frequent anomalies are cranial, but extracranial findings may also relate to viral infection. Cranial anomalies, especially microcephaly and ventriculomegaly, are associated with a poor postnatal prognosis [5]. Congenital CMV infection is considered the most common non-genetic cause of fetal sensorineural hearing loss [6]. Both the level of fetal transmission and severity of the disease can be lowered with proper intrapartum and postpartum therapies, such as immunoglobulins and antiviral administration to the mother and fetus during pregnancy and in the postpartum period [7]. This review aimed to shed light on the state-of-the-art methods for the prevention, prenatal diagnosis, and management of congenital CMV infection.

## 2. Materials and Methods

A literature review was conducted in the PubMed and EMBASE databases to select full-length English articles published in the last fifteen years up to August 2022. Both adult and congenital CMV infections were included to properly assess the diagnosis and management of the disease. The congenital infection of the fetus is challenging to diagnose, and protocols have been continuously designed and adjusted to achieve an accurate and early diagnosis. Fetal involvement, evaluated using imaging techniques or directly from amniotic fluid and fetal blood, and the prevention of postpartum sequelae are highly important.

Among the research strategy keywords, we included cytomegalovirus, immunoglobulins, congenital CMV infection, antiviral treatment, and CMV ultrasound diagnosis.

## 3. Results

### 3.1. Background

Cytomegalovirus is a virus that belongs to the *herpesviridae* family of viruses and only develops within human cells. The largest and most complex of its family, the human herpes virus 5 has a 220 nm diameter and a genome composed of 235,000 double-stranded DNA macromolecules. Its DNA assembles as circular DNA and replicates best within human fibroblasts [2].

### 3.2. Epidemiology

CMV infection epidemiology is reported to differ among populations and geographical areas. Although often documented as around 60%, the incidence varies from 40% to 100% in the populations of developing countries [1,8]. Viral infection may occur from a positive and contagious patient through close contact (urine, saliva, and other fluids), the transfer of blood products; sexually; transplacentally; perinatally; and during breastfeeding.

Like other herpes viruses (such as herpes simplex type 1 and 2; varicella zoster; and human herpes viruses 6, 7, and 8), CMV is known to be associated with infections that have a persistent and latent pattern [2]. Following primary infection, associated or not with clinical manifestations, CMV reinfection with other related viral strains is possible at any moment during one’s lifetime. Reactivation is particularly associated with other medical conditions or diseases that suppress the host’s immune system [1,9,10].

### 3.3. Clinical Manifestation

CMV is a commonly spread virus associated with heterogeneous clinical manifestations among infected patients. Usually, the infection is acquired during childhood. Patients may develop symptoms within 9 to 60 days following primary infection. Human salivary glands are frequently involved, and the patient secretes and transmits the virus through saliva and other body fluids. The clinical disease may vary from being non-symptomatic or displaying mild symptoms to causing life-threatening medical conditions when patients are immunocompromised [11].

The symptoms can be absent or mild, and they include a fever, commonly of unknown origin; rashes; pharyngitis; and lymphadenopathy. Paraclinical findings may show leukocytosis, anemia, thrombocytopenia, increased liver enzymes, and occasionally abnormal immune antibodies [12]. CMV infection can also cause pneumonia, encephalitis, neuropathy, hepatitis, posterior poll eye damage, digestive and urinary tract disease, and other conditions [1,2]. Severe symptoms and organ damage are often diagnosed in immunosuppressed patients. The condition usually affects patients with HIV disease and organ or bone marrow transplant recipients, and, in those cases, CMV reactivation can lead to life-threatening conditions and organ failure [13,14].

### 3.4. Diagnosis

The diagnosis of CMV infections is rarely required in the common population, but it is necessary during pregnancy and in immunosuppressed patients. Multiple testing methods are nowadays available, such as antibody serum detection, direct detection from human fibroblast cultures, and quantitative real-time polymerase chain reaction (PCR) for the detection of viral DNA [2].

Antibody serum detection is the most common type of diagnosis. Primary infection is characterized by the presence of immunoglobulin M (IgM) antibodies in the patient’s serum, which are detected as early as four weeks following primary infection and may persist for a maximum of 20 weeks. Blood viral DNA is commonly found in positive patients together with IgM antibodies [8]. The confirmation of diagnosis may require the detection of DNA using PCR techniques. This is especially important in pregnant women and during breastfeeding, because of probable fetal transmission.

The postinfection antibodies that persist throughout the lifetime are immunoglobulin G antibodies. Natural immunity following the disease does not protect against reinfection. Cell-mediated immunity is related to reinfection or reactivation through CD4 and CD8 lymphocytes [1]. These CMV-related immune cells play a crucial role in the reactivation of the disease, especially in immunosuppressed patients [10].

### 3.5. Management and Treatment

The management of CMV primary infection or reinfection/reactivation in immunosuppressed patients depends on the immune status and the associated symptoms. Treatment is rarely required in immunocompetent patients. For HIV patients or those with other immune-suppressing medical conditions, both prevention and treatment are recommended [14].

Treatment protocols for moderate and severe forms of the disease include antiviral agents, such as valganciclovir, ganciclovir, and foscarnet [11]. This therapy is considered for immunocompromised patients. For healthy patients, medication is only used to address clinical symptoms, because the infection has a good prognosis [15].

In serious infections, both antiviral therapy and organ-specific medication are not often associated with full recovery, depending on the patient’s concomitant medical conditions and immune status. Immunocompromised patients with a severe form of the disease and multiple organ involvement have a high risk of morbidity and mortality. In those cases, the reactivation of CMV infection is very common [16,17].

### 3.6. CMV Infection in Pregnancy

In addition to adult infection, the CMV virus is one of the most common congenital infections that complicate pregnancies and the well-being of newborns. The incidence is reported to be around 1% but varies from 0.2 to 6.1% of live births. Significant differences are reported worldwide [7,18]. In western Romania, in 2021, the incidence of seropositive CMV was between 91.8 and 94%. The IgM prevalence was approximately 0.3%, and the seronegative prevalence was 0.7% among young reproductive-age women. Higher incidence was reported in urban areas, namely 0.9%. These two groups are likely to be the most susceptible to primary infection and complications during gestation and the postpartum period [19].

The prevalence of the infection, a proper diagnosis, and management during pregnancy determine the fetal outcome and prognosis [20].

### 3.7. Transmission of the Infection

It is reported that 80% to 95% of pregnant women with a primary infection develop no symptoms suggestive of a CMV infection, while the rest have mild, influenza-like symptoms [7]. The disease should be suspected and diagnosed as early as possible during gestation because of the potentially poor fetal outcome. When infected, the mother can vertically transmit the virus to the fetus through the placenta or to the newborn during labor and breastfeeding [21].

Though primary infection is associated with the highest risk of fetal involvement and reinfection, secondary infections have also been investigated. The rate of transmission to the fetus in pregnant women with a secondary infection is unknown, though it is thought to be 0.2 to 2% [4]. Even in developed countries, the diagnosis of a non-primary infection is not certain. Proper evaluation and diagnosis methods should be established to distinguish between primary and secondary infections and further fetal involvement [22].

During pregnancy, the transplacental transmission rate varies from 30 to 70% of cases [7]. These differences are related to both the type of maternal infection and the age of gestation. The infection risk increases with the gestational age. In the periconceptional period and the first trimester, the risk is the lowest (17 to 35%), but it reaches as high as 72% in the second and third trimesters [3,23]. Contrary to the infection rate, fetal symptoms and clinical disease appearance considerably decrease throughout pregnancy. Long-term postpartum CMV was more likely to develop when infection occurred early during pregnancy (in up to 20% of cases), compared to 6% in the second trimester, and even lower later on during gestation [24].

### 3.8. Diagnosis of Fetal Infection

Pregnant women are often asymptomatic, and the transplacental transmission rate varies with gestational age; hence, the mother and the fetus must be thoroughly evaluated [21]. Guidelines for CMV detection in pregnancy recommend first-trimester screening for specific antibodies, as congenital CMV infection is related to various forms of fetal organ damage [5]. The first-trimester screening of CMV infection is appraised within the TORCH complex, together with toxoplasmosis, rubella, herpes simplex, and other infections. The newborn is postnatally evaluated for CMV-specific anomalies. The infection-related sequelae may often be permanent and severe, even though they may sometimes not be present at birth, but develop later in life [25,26].

### 3.9. Maternal Serum Testing

A CMV congenital infection is still difficult to diagnose. Considering the non-specific symptoms, serum antibodies should be tested [27]. The clinical manifestation is often associated with the flu or mononucleosis symptoms, such as fever, fatigue, cervical lymph nodes, and myalgia [3]. Paraclinical changes may reveal elevated lymphocytes and abnormal liver function. The clinical symptoms are more commonly present during primary infection, and less likely in reinfections or the reactivation of the disease. If maternal infection is suspected, additional testing is required, such as antibody avidity tests, to establish the moment of infection and evaluate the risk of fetal infection [4,28].

**Figure 1:**
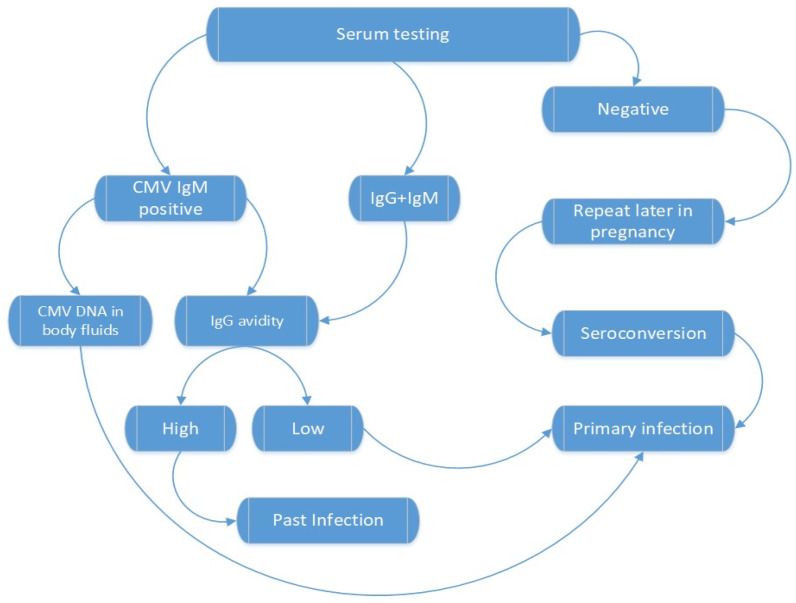
Maternal diagnosis of CMV infection [4].

Both CMV-specific IgM and IgG antibodies must be tested in the maternal blood. Serum CMV IgM is present in both primary and secondary forms of the disease and represents a diagnosis of maternal CMV infection (Figure 1) [5,23].

It is often difficult to determine the moment of primary infection, because the virus can be active for months or sometimes years. Additionally, due to additional medical conditions, the virus can reactivate, or the pregnant woman can be infected with a different viral strain, so the fetus can be vertically infected even if the mother was immune periconceptional. IgM testing during pregnancy may result in false-positive results, especially due to cross-reactions with other viruses, such as Epstein–Barr, herpes simplex, and varicella-zoster, or autoimmune diseases [25,26]. If IgM antibodies are present, DNA PCR detection, IgG, and IgG antibody avidity must be investigated [29].

When seronegative pre-conceptionally or at first-trimester screening, patients should later be tested for both IgM and IgG antibodies. If IgM and IgG antibodies appear later during pregnancy, they can be used to diagnose a primary maternal infection and potential fetal infection [30]. Further testing for CMV IgG avidity is required. Low avidity, less than 35–50%, is indicative of recent infections, while high avidity, more than 50–65%, indicates past infections. Moderate-to-high-avidity antibodies are also indicative of a primary maternal infection if detected after 21 weeks of gestation [4]. The timing of antibody testing is crucial in fetal prognosis and evaluation. Notable differences between laboratory tests and kit thresholds may appear. Primary and secondary maternal infections, but also fetal involvement, could be differently interpreted [31]. High-avidity IgG during the first trimester is related to past infection, whereas low-avidity antibodies, especially with long persistence, are associated with IgM and can lead to a false diagnosis of primary maternal infection. Additionally, the rapid increase in the avidity of IgG is associated with a higher risk of intrapartum fetal infection [32].

Many researchers have tried to differentiate primary from secondary/reactivated infections in relation to fetal and congenital forms of the disease. Serum testing refers to more specific and neutralizing antibodies, but they are not routinely available [33].

### 3.10. CMV Confirmation

Following the evaluation of maternal serum antibodies, the infection should be confirmed by CMV detection, either from blood or other body fluids. This is a means to diagnose with certainty an active maternal infection, but the sensitivity decreases within the first month after infection [34]. Urine, saliva, vaginal secretions, blood, and amniotic fluid cultures can be used to isolate CMV, but blood and urine analyses are performed more often. The urinary excretion of CMV is associated with an ongoing, active infection with an additional high risk of fetal infection [35,36]. The presence of CMV DNA in the blood is more frequent in primary infections, but it can also be present, at a decreased rate, in non-primary infections [37].

CMV infection diagnosis can be established by direct viral detection within a cell culture, most frequently from a urine sample. Although this method was standard for a long period of time, the diagnosis protocol had to be updated due to the existence of multiple significant limitations. For instance, the time required for the virus to replicate in the culture may extend up to 30 days after inoculation, especially if the viral load is low [38]. Additionally, there is a risk that the sample’s microbial flora could significantly influence the viral replication and final diagnosis. Besides standard cultures, the direct isolation of the virus using the shell vial method offers rapid detection within 24 h. This method has a higher sensitivity than the standard culture method, but studies recommend that both methods should be used for a precise diagnosis [4,39].

Currently, the standard and most frequently used diagnosis method is molecular DNA PCR detection. With high performance, specificity, and sensitivity, real-time PCR does not have the limitations of the previously mentioned diagnosis methods, such as the risk of sample contamination. Additionally, kits for diagnosis are easily available in different laboratories, low-cost, and do not require special storage and transportation conditions [3,40,41].

Following infection, patients develop a specific T-cell immunity that can be a crucial prognosis factor regarding fetal infection. Several methods are used to determine CMV cell-mediated immunity. A specific CD24+ T-cell is responsible for CMV immunity and is important for both adult infection and fetal involvement. A non-reactive decreased lymphoproliferative immune activity and a high number of specific T-cells are frequently associated with a high risk of fetal infection during pregnancy [42,43].

### 3.11. Fetal Diagnosis

When a maternal diagnosis is suspected or confirmed, fetal infection should be properly evaluated. Abnormal fetal findings are difficult to establish and commonly unspecific; moreover, newborns may appear normal at birth but develop abnormalities associated with a congenital CMV infection later in life [44].

**Figure 2:**
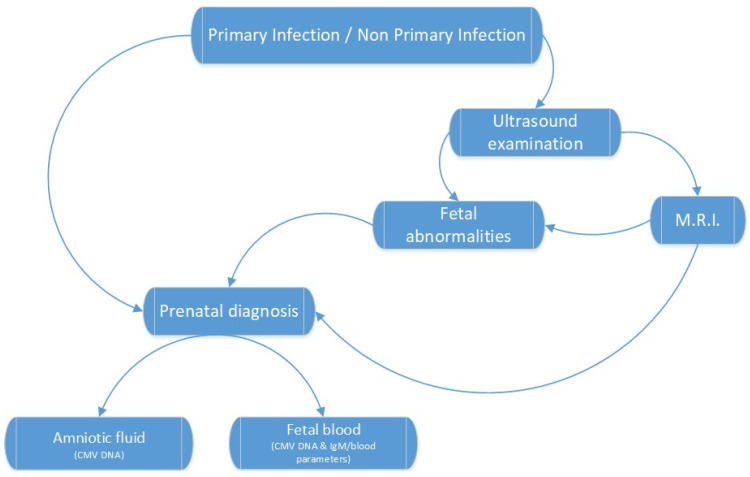
Fetal diagnosis of CMV infection [4].

A fetal CMV infection is often difficult to diagnose; hence, various methods, invasive and non-invasive, are available, as shown in Figure 2 [4]. Direct detection using amniocentesis and cordocentesis has a high sensitivity and specificity, but it is an invasive method. Ultrasound and magnetic resonance imaging are non-invasive, but they have a low specificity and require specialized medical personnel to properly assess abnormal findings [38,45].

### 3.12. Amniocentesis and Cordocentesis

The confirmation of fetal involvement requires the direct detection of CMV in an amniotic fluid culture. Together with the evaluation of blood parameters acquired through cordocentesis, this method has the highest prediction ability, but both methods are avoided because of the associated procedure risks [46].

Amniotic fluid diagnosis is performed by the direct detection of the virus in a specimen culture, or by real-time PCR. The procedure must be executed as early as at 20 weeks of gestation and re-examined at 6 to 8 weeks following suspected maternal CMV infection. Timing is mandatory and related to the renal viral excretion of the infected fetus into the amniotic fluid [47]. Most false-negative diagnoses are related to early investigations, less than 8 weeks since maternal infection and before 18 weeks of gestation. Besides viral presence, the viral CMV load detected in amniotic fluid is directly proportional to and associated with fetal involvement and the severity of the infection. A result above 105 genome equivalents suggests a severe form of fetal disease, while a value below 103 most probably excludes a significant infection and future symptomatic disease in the fetus [4,48].

Additionally, researchers have tried to identify and detect specific amniotic fluid proteins that can predict fetal disease. The presence of a series of proteins responsible for inflammatory and immunologic response is identified in the amniotic fluid of severely infected fetuses. The abnormal amount and preponderantly inflammatory response found in the maternal–fetal barrier, amniotic fluid, and placental sample is indicative of a severe form of the disease with neurologic and cerebral damage [49,50].

Cordocentesis can be used to assess various blood parameters and predict fetal disease, but it carries a greater risk compared to amniocentesis, with up to 1.9% fetal loss. IgM levels, viral DNA, thrombocytes, and other blood elements can be directly analyzed [51,52]. Additionally, cordocentesis can be used to determine a variety of blood infections and hematological abnormalities. Despite the very high specificity of the procedure, DNA detection from a blood sample through cordocentesis is rarely performed [53]. However, amniocentesis has a higher sensitivity when compared to cordocentesis. Nevertheless, cordocentesis remains useful when the viral load is increased and thrombocytopenia is diagnosed, as it can more accurately predict both fetal infection and postnatal outcomes [54].

### 3.13. Fetal Imaging Diagnosis

Ultrasound fetal imaging, sometimes associated with complementary MRI, is the most common procedure for evaluating fetal growth, genetic diseases, and organ morphological anomalies [55]. Currently, congenital CMV diagnosis is assessed using imaging procedures, although they have low specificity and precision and frequently depend on medical personnel with considerable experience. Following maternal and fetal infection, ultrasounds should be performed with a frequency of every 3 to 5 weeks to assess fetal abnormal imaging findings [56]. Ultrasound and MRI have been helpful in determining both congenital CMV diagnosis and fetal prognosis [7]. Whenever a lesion is suspected, a targeted fetal ultrasound or MRI should be performed, especially in the third trimester, when they have the highest sensitivity [57]. MRI imaging has a higher sensitivity than ultrasound, but the procedure is expensive, difficult to evaluate, and requires specialized medical personnel. Nevertheless, MRI may predict fetal involvement and exclude severe forms of the disease if findings are absent or subtle [58].

Congenital CMV impacts fetal central nervous system (CNS) development and hence leads to extracranial abnormalities, sensorineural hearing loss, and placental and amniotic fluid anomalies [59].

**Figure 3:**
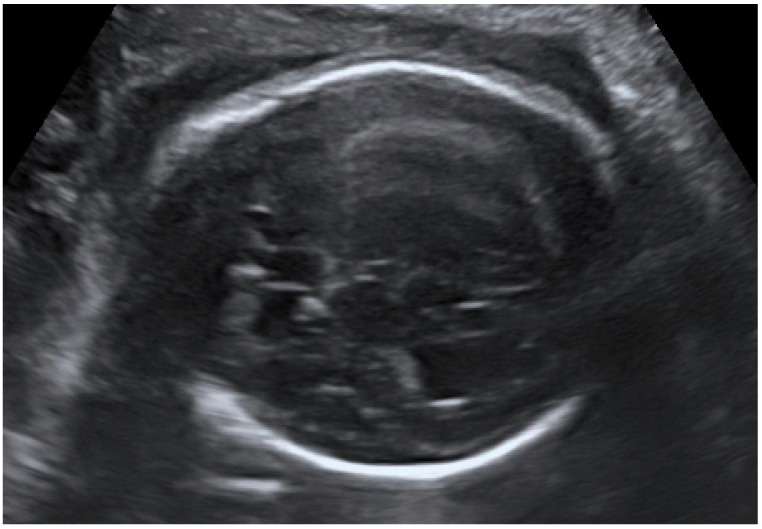
Dilation of the anterior and posterior horns of the lateral and third ventricles.

**Figure 4:**
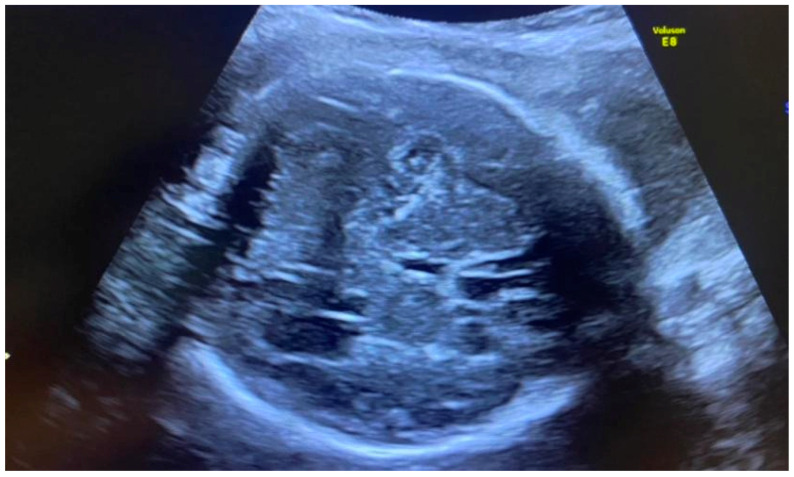
Intracranial calcifications; head circumference below the 3rd percentile—microcephaly.

Congenital CMV fetal infection may be associated with a variety of CNS abnormalities. Those may be severe or mild; appear due to the early inflammatory, destructive, and obstructive processes of the brain infection; and directly influence fetal prognosis [60]. Ventriculomegaly (more than 15 mm), microcephaly (a decrease of less than two standard deviations), high echogenicity in the periventricular areas, and hydrocephaly may appear as severe intracranial signs. Increased cisterna magna (more than 8 mm), vermian hypoplasia, periventricular cysts, agenesis of the corpus callosum, lissencephaly, and porencephaly are often associated with congenital CMV infection [4,5,61]. Of these, ventriculomegaly and microcephaly are associated with the worst fetal prognosis. The mild cerebral findings related to fetal involvement include ventriculomegaly enlargement (10 to 15 mm) (Figure 3), intracranial calcifications (Figure 4), cysts of the choroid plexus, subependymal cysts, and intraventricular synechiae [62,63].

**Figure 5:**
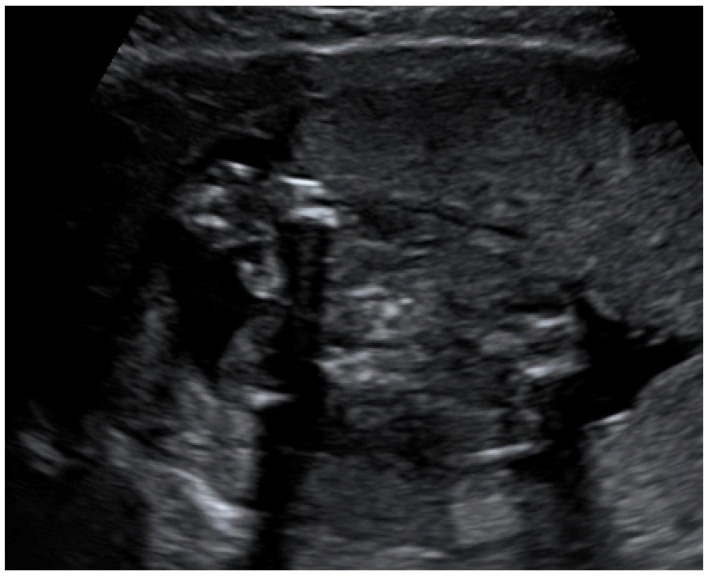
Bowel hyperechogenicity.

Whole fetal organs may be directly or indirectly affected by viral infection. Most of the time, the imaging signs are unspecific and may be associated with causes other than congenital CMV, genetic or not. Digestive tube abnormalities can be related in particular to the hyperechogenicity of the bowel (Figure 5), intrahepatic calcifications, and hepatosplenomegaly, as well as pleural effusion, fetal edema, ascites, or hydrops [64,65]. Splenomegaly is considered a common ultrasound finding related to congenital CMV infection. Researchers found that the examination of the splenic artery by Doppler evaluation may be used as a non-invasive marker for congenital CMV, probably related to impaired arterial blood flow [66]. Congenital infection may influence cardiac development, and signs such as cardiomegaly, the effusion of liquid in the pericardium, and calcifications may be highlighted [5].

Placental involvement can be observed before fetal anomalies are detected and is characterized by a heterogenous appearance, thickness (placentomegaly), and calcifications. These can be diagnosed starting from 12 weeks following maternal infection, while the time frame for fetal abnormal imaging findings is much longer, as they appear after 20 weeks of gestation [67,68]. Placental involvement is most often associated with fetal growth restriction [69]. Additionally, ultrasound can diagnose oligohydramnios or polyhydramnios [70]. It is believed that despite the extracranial findings, the fetus can recover from a congenital infection if CNS abnormalities are not present [71].

Ultrasound and MRI findings must be complemented with laboratory parameters from amniotic or fetal blood samples via cordocentesis to assess fetal involvement, prenatal diagnosis, and outcome [72]. The presence of CMV IgM and beta-2 microglobulin, low platelet levels (less than 100,000/mm^3^), increased values of hepatic enzymes (alanine aminotransferase above 80 IU/mL), and an increased level of direct bilirubine (more than 4 mg/dL) are associated with the presence of symptoms at birth and poor fetal prognosis [56,59,73].

### 3.14. Prevention and Management of CMV Infection

The congenital infection of the fetus can be prevented by assessing the status of the infection, transmission, and intrauterine treatment [74]. In selected cases, when fetal infection and disease are highly probable, the termination of the pregnancy could be taken into consideration. Patients should be properly informed about the available treatment, its benefits and risks, and related fetal complications. It is difficult to accurately predict fetal involvement and related permanent sequelae.

Seronegative patients should avoid infection periconceptionally and during pregnancy. Proper hygiene significantly decreases the risk of potential infection [3]. In addition to general hygiene measures, a potential vaccine against CMV could also be a measure of prevention for both primary and non-primary infections in at-risk populations or during pregnancy. Immunosuppressed patients and organ recipients will probably benefit most from a CMV vaccine, being at risk of a severe form of the disease caused by a CMV primary infection or reactivation. Unfortunately, more studies are required for a vaccine to be developed with sufficient efficacy against CMV. Currently, no approved CMV vaccine is available for medical use [75,76,77].

The transmission of the infection to the fetus in a seropositive pregnant woman is considered when a diagnosis of CMV infection is suspected or established. Usually, a maternal infection is equal to a fetal infection [78], but the risk of intrauterine symptomatic disease is highly dependent on the age of the pregnancy. Additionally, fetal disease and abnormalities are closely related to gestational age [79]. There is no consensus regarding the treatment of a congenital infection, despite the increasing methods of diagnosis that have been developed. Both CMV immunoglobins and antiviral drugs are available and may be included in the treatment regimen. They should be recommended and administered in selected cases, following a proper risks and benefits evaluation and patient approval.

When the maternal infection has been diagnosed periconceptionally, in the first trimester, or in the early second trimester, the risk of fetal infection and disease can be decreased by administering hyperimmunoglobulin [80]. Studies report that high-avidity immunoglobins administered to a seropositive patient decrease the risk of fetal infection from 40% to 16% and from 44% to 30%, respectively [80,81]. Others reported a transmission rate of 23% in the presence of specific treatments. The administration of hyperimmunoglobulin in the first 20 weeks of gestation is considered beneficial, particularly in CMV primary infections, having demonstrated a decrease in fetal disease cases in terms of infection and clinical abnormalities. After 20 weeks of gestation, there is no consensus on the benefit of treatment administration [81,82].

When fetal transmission is suspected, management should be focused on avoiding symptomatic conditions and decreasing fetal lesions and organ involvement [83]. Following transmission and amniotic viral detection, immunoglobulin treatment significantly prevents and lowers the chances of fetal clinical disease [84]. For best results, the administration of hyperimmunoglobulin via cordocentesis should be preferred to maternal perfusion because of the low concentration of immunoglobin achieved through placental passage. These procedures are not fully standardized, and some believe that further studies should be performed to determine the route of administration, frequency, and doses [85].

Following the diagnosis of fetal involvement, the administration of antiviral agents can also be considered [86]. Antiviral treatments in pregnancy are carefully administered, considering both toxicity and drug efficacy. Focus has been set on valaciclovir, which has high bioavailability and amniotic concentrations that are sufficient when administrated orally. A dose of 8 mg daily has been proven safe and efficient in first-trimester maternal primary infections, reducing the risk of fetal disease compared to a placebo. Administered early during gestation after maternal infection, it can reduce both the termination of pregnancy and congenital CMV infection. Unfortunately, the efficacy was non-statistically significant in the case of the periconceptional infection of pregnant woman [87,88].

### 3.15. Fetal Outcome and Prognosis

Congenital CMV infection is one of the most frequent viral fetal infections and leads to a series of CNS and extracranial anomalies associated with long-term deficiencies in the patients involved [89]. Additionally, it is the most common non-genetic cause of neurosensorial hearing loss and has a severe impact on neurological development [90,91].

As well as other congenital infections, CMV can be associated with an increased risk of miscarriage. This is especially related to CMV placental impairment. There are several mechanisms that impact the normal development of the placenta. The timing of viral infection is crucial in relation to future placental damage. The early infection of the trophoblast can determine pregnancy loss or, more frequently, intrauterine growth restriction [92].

The most common clinical findings at birth are growth restriction or small for gestational age, jaundice, CNS anomalies, hepatosplenomegaly, and blueberry muffin rash. It is of utmost importance that the diagnosis of a congenital infection is carried out by the 21st day of life, usually by DNA detection or PCR from body fluids such as urine, saliva, or cerebrospinal fluid [56,93].

Only 10 to 15% of contaminated fetuses present abnormalities at birth, but up to 25% are diagnosed later in life with a CMV-related disease. From the high proportion of asymptomatic newborns, 5–15% present with several sequelae related to CMV infection, especially neurosensorial hearing loss [94,95]. If symptoms are clinically detected, sequelae are more frequent, in approximately 50% of postpartum cases within the first year of life [96]. Children present hearing loss, cerebral palsy, vision disorders, and intellectual disabilities, as well as other specific manifestations related to previous intrauterine organ involvement [97,98]. Congenital CMV is the leading cause of fetal non-genetic sensorineural hearing loss. It can develop uni- or bilaterally and is diagnosed in up to 30–40% of symptomatic children and 5–10% of asymptomatic children at birth. Unfortunately, the hearing damage is often progressive following postpartum diagnosis, and 30% of these cases develop deafness later in life. Newborns should be systematically evaluated at birth regarding their hearing, especially because congenital CMV often requires a cochlear implant to restore function [99,100].

Given the permanent sequelae, children can develop disabilities due to CMV infection, but if the infection is diagnosed at birth, then antiviral treatment is indicated [101]. Because of its toxicity, the treatment is not routinely recommended to infants, and cases should be carefully selected. The therapy recommends six weeks of intravenous ganciclovir, which has been proven to interrupt hearing loss and benefit deteriorated neurological and psychological functions. It is recommended that the six weeks of treatment are followed by another six months; some suggest 12 to 24 months of oral valganciclovir to maintain the beneficial effect of the antiviral treatment of CMV-related sequelae [102].

Newborns can be infected postpartum through direct contact with body fluids while breastfed and, for premature births or those with other medical conditions, via blood transfusions from a CMV-infected donor. Depending on prematurity and associated diseased, they can develop severe forms of CMV infection [103]. The associated septic symptomatology, hepatosplenomegaly, impaired liver function, pneumonia, hemolytic anemia, thrombocytopenia, and lymphocytosis have a mortality of up to 20% and serious long-term sequelae. Antiviral therapy decreases the viral load and associated organ disease [104,105].

## 4. Conclusions

Cytomegalovirus infection is not to be feared in the general population, despite the high prevalence among children and adults. Unfortunately, the infection and related complications may be associated with severe medical conditions in immunocompromised patients and during pregnancy and the postnatal period. The infection is a time-dependent disease related to gestational age. The most severe anomalies are encountered when infection develops periconceptionally or early during gestation. Major progress has been achieved in accurately determining both maternal and fetal infection, as well as fetal disease. Unfortunately, even if a diagnosis has been confirmed, there are still challenges to precisely determining fetal disease and prognosis. Congenital CMV is responsible for cranial and extracranial anomalies, associated with long-term deficiencies, and it is the most common non-genetic cause of fetal sensorineural hearing loss. Considering the fetal sequelae, methods to decrease fetal involvement, such as hyperimmunoglobulins and antiviral administration, are crucial immediately after diagnosis and early during pregnancy. Postnatal evaluation is also important because many newborn infants with congenital CMV may be asymptomatic at birth and permanent sequelae can develop within the first year of life. Managing fetal CMV infections is complex and concentrates on limiting disease extension. Screening and hygiene measures are the best methods to prevent congenital CMV and associated complications.

## Data Availability

Not applicable.
